# Energy-Efficient Biochar Activation in a Fluidized Bed Reactor Using CO_2_–Air Mixed Atmospheres

**DOI:** 10.3390/molecules31040724

**Published:** 2026-02-20

**Authors:** Reyhane Aghaei-Dinani, Neda Asasian-Kolur, Michael Harasek

**Affiliations:** Institute of Chemical, Environmental and Bioscience Engineering, Technische Universität Wien, Getreidemarkt 9/166, A-1060 Vienna, Austria; reyhane.aghaei@tuwien.ac.at

**Keywords:** biochar activation, CO_2_–air co-activation, fluidized bed reactor, pore structure development, energy-efficient process, thermodynamic analysis

## Abstract

Biochar activation is critical for producing high-performance adsorbents; however, conventional activation methods are energy-intensive and difficult to control, particularly when air is used as an activating agent. This study investigates CO_2_–air co-activation in a laboratory-scale fluidized bed reactor as an energy-efficient alternative. Experiments were conducted at 750–850 °C under varying gas flow rates with a constant CO_2_/O_2_ ratio. Optimal properties were achieved at 800 °C and 0.2–0.3 L/min CO_2_, yielding a maximum BET surface area of 479 m^2^/g, a micropore contribution of 42%, and controlled carbon conversion (~25–35% yield). Aspen Plus equilibrium simulations also confirm that CO_2_-only activation remains endothermic (heat duty up to +0.07 kW), air-only activation becomes strongly exothermic (down to −0.13 kW), while the CO_2_–air mixture exhibits near-thermoneutral to mildly exothermic behavior (+0.13 to −0.10 kW), thereby reducing external energy demand potentially by approximately 60–70% compared with CO_2_-only activation and significantly improving process stability. These results demonstrate that CO_2_–air co-activation offers a practical route to produce high-quality activated biochar with controlled porosity and improved energy efficiency.

## 1. Introduction

Biochar is a sustainable, carbon-rich material produced through the pyrolysis of biomass, a process in which organic matter is thermally decomposed in the absence of oxygen. Derived from sources like agricultural residues, forestry by-products, and biowastes (e.g., sewage sludge), it is both accessible and cost-effective. Often generated as a byproduct in industries such as biomass power, biofuel production, waste management, and agricultural processing, biochar offers diverse applications and significant environmental benefits, including mitigating climate change, soil degradation, and pollution [1]. Depending on operating conditions and system design, thermal gasification systems can generate high-quality biochar as a valuable by-product with significant application potential and economic relevance, as highlighted in recent technology reviews [2].

A key advancement in biochar technology is the activation process, which enhances its porosity. Activation methods comprise both thermochemical approaches (e.g., steam or CO_2_ activation and chemical activation using acids, alkalis, or metal oxides) and non-thermal physicochemical techniques such as microwave, electrochemical, ultrasonic, and plasma treatments [3]. Physical activation of biochar is a process in which high-temperature exposure to oxidizing gases removes volatile carbon components, opens closed pores, creates new pores, and enhances diffusion within the pore structure. This process converts biochar into activated carbon with improved adsorption capabilities. Activated biochar can efficiently remove pollutants from air and water by adsorbing contaminants such as heavy metals, organic compounds, and dyes [4].

Among the commonly employed physical activating agents (CO_2_ and H_2_O), CO_2_ is favored for its ability to form highly microporous structures, offering precise pore control due to its endothermic reaction, though it demands significant energy [5]. Conversely, steam activation efficiently develops and enlarges micropores owing to its smaller molecular size, which enhances penetration and accelerates reaction times, but similarly requires external energy [5,6,7]. However, both activating agents are employed at comparable levels due to the unique pore structures they generate in carbonaceous materials. Depending on the size distribution of adsorbate molecules and their phase behavior, varying degrees of porosity, including both micro- and mesoporosity, may be desirable. Asasian Kolur et al. [8] showed that the choice of physical activating agent strongly governs biochar pore development, kinetics, and process efficiency, with steam being markedly more reactive than CO_2_, yielding higher surface area and mesoporosity, and requiring much lower activation energy (64.8 vs. 117.2 kJ/mol). They further demonstrated that integrating carbonization and activation into a single streamlined process can reduce total energy consumption by approximately 30% while enhancing product performance [8].

In contrast to CO_2_ and H_2_O, activation with O_2_ involves exothermic reactions with carbon and therefore requires strict temperature control to prevent combustion and minimize ash formation, making the process more challenging. However, air, as a low-cost oxygen source, can serve as an energy-efficient activating agent [6,9]. Uncontrollable burn-off and low product yields, along with the lower surface areas and pore volumes of carbons produced with oxygen compared to steam or CO_2_ activation, have been reported in early studies. Levan et al. [10] reported minimal changes in texture and porosity when using air or diluted (10%) oxygen in N_2_ for coal-derived carbons [10,11]. Allen reported that activating anthracite in 21% oxygen produces carbons with poor textural properties, reinforcing the general trend that activating effectiveness decreases in the order: steam > CO_2_ > O_2_ [11].

Peredo-Mancilla et al. [12] showed that H_2_O activation of olive stones produces a more accessible pore network than CO_2_, while also generating oxygen-containing surface groups that enhance CO_2_ adsorption via acid–base interactions [12]. Similarly, Rodríguez-Reinoso et al. [5] demonstrated that CO_2_ primarily promotes the formation and widening of narrow micropores, whereas H_2_O induces early micropore widening and increases meso- and macroporosity, improving pore accessibility and diffusion; these intrinsic differences persist even at comparable gasification rates [5]. In contrast, Heras et al. [13] reported that air is a much more reactive gasifying agent than CO_2_, leading to rapid burn-off at temperatures above 550 °C [13]. Bosch et al. investigated one-step activation of waste wood in a fluidized bed reactor using CO_2_ and H_2_O separately at 750–850 °C and 5–20 min. The maximum specific surface area obtained with CO_2_ activation was approximately 620 m^2^/g at 850 °C and 12.5 min, whereas H_2_O activation achieved a higher value of 822 m^2^/g at 850 °C and 20 min [14].

The combination of activating agents (especially H_2_O and CO_2_) has also been studied in a few studies to yield superior pore structures and surface areas compared to single-agent activation. For instance, Cheng et al. [15] investigated the co-activation of willow wood with biochar and found that the presence of H_2_O and CO_2_ during activation facilitated additional pore generation by intensifying the gasification process. The synergetic effect of using H_2_O and CO_2_ together resulted in activated carbons with higher surface areas and enhanced porosity compared to single-agent activation [15]. Li et al. [16] also used a mixed air/nitrogen atmosphere in combination with an alkali activating agent and demonstrated that air significantly improves mesopore development compared to activation in pure nitrogen, particularly when using alkali (NaHCO_3_) [16]. Gurtner et al. [17] demonstrated that specific surface areas above 800 m^2^/g can be achieved using a sequential H_2_O-CO_2_ activation process; however, this approach resulted in severe burn-off and low carbon yields (≈38.6%) [17,18]. Although they also examined CO_2_-only and air–CO_2_ activation, meaningful comparison between these systems is limited due to substantially different operating conditions.

Previous studies have demonstrated that combining CO_2_ and O_2_ during char formation from biomass can significantly enhance char reactivity and pore development compared with single-gas atmospheres. Hanaoka et al. [19] investigated biomass char produced under N_2_/CO_2_/O_2_ atmospheres and showed that chars formed with approximately equal fractions of CO_2_ and O_2_ exhibited the highest BET surface area and CO_2_ gasification reactivity among the conditions studied. This behavior was attributed to the synergistic interaction between partial oxidation and CO_2_ gasification, which promoted pore formation and generated a more amorphous and reactive carbon structure [19]. These findings suggest that mixed CO_2_–O_2_ environments can create highly reactive carbon materials by coupling endothermic and exothermic reactions, providing a promising basis for developing efficient and controllable biochar activation strategies. The work by Gurtner et al. [20] demonstrated the feasibility of physically activating gasification char at pilot scale using air combined with CO_2_ and/or H_2_O. The process employed a continuously operated screw reactor integrated into an industrial wood gasification plant. Activation with pure air, air–CO_2_, air–H_2_O, and air–CO_2_–H_2_O atmospheres was systematically compared. Among the investigated activation routes, air–CO_2_ activation yielded the highest pore development, achieving BET surface areas of up to 661 m^2^/g corresponding to a relative increase of up to 146%, but was accompanied by increased carbon conversion to gas. Pure air activation showed the highest energy efficiency due to its self-sustained operation, though with lower BET surface areas (up to 478 m^2^/g). Air–H_2_O activation resulted in high porosity (up to 605 m^2^/g) but was limited by reduced reactor temperatures caused by endothermic reactions. Overall, the results indicate a clear trade-off between pore development, carbon conversion, and energy efficiency across the activation processes [20].

The choice of activation reactor is crucial in determining the efficiency and quality of activated carbon production. Al-Farraji et al. compared the pyrolysis of olive kernel biomass in a thermogravimetric fluidized bed reactor (TGFBR) and a conventional thermogravimetric analyzer (TGA) which operates as a fixed bed tractor, showing that TGFBR, with higher heating rates and larger sample sizes (60 g vs. 20 mg), exhibited faster pyrolysis and lower activation energy (67.4–60.8 kJ/mol vs. 74.4 kJ/mol in TGA) [21]. Kinetic analysis revealed two-dimensional diffusion in TGA, while TGFBR showed three-dimensional diffusion at higher temperatures (500–660 °C). Particle size affected reaction rates in TGFBR but had minimal impact in TGA. Overall, TGFBR better simulates industrial-scale pyrolysis, while TGA is suited for kinetic studies.

The superior heat and mass transfer of fluidized bed reactors compared to fixed bed reactors is well known. Recent CFD simulations of fluidized-bed fast pyrolysis demonstrate that reactor hydrodynamics is a key parameter governing product distribution and conversion efficiency [22]. In the context of biochar combustion, Ferreira et al. reported that although both reactor types benefit from higher operating temperatures, fluidized beds consistently outperform fixed beds due to enhanced gas–solid interactions. These interactions reduce oxygen diffusion limitations and interparticle competition, allowing reaction kinetics to dominate. In contrast, fixed bed reactors experience pronounced diffusion constraints—quantified through interparticle competition and constriction factors- which hinder oxygen transport and slow the overall combustion process [23].

The Management Center Innsbruck (MCI: Internationale Hochschule GmbH), through its spin-off SynCraft^®^, has developed a floating fixed-bed gasification reactor capable of converting low-quality wood chips, including bark and fines, into renewable energy, with biochar produced as a by-product [24]. The present study investigates a hybrid activation approach that combines air and CO_2_ to activate this biochar in a fluidized-bed reactor, aiming to couple exothermic and endothermic reactions by integrating limited air with CO_2_ activation. The objective is to examine the effects of two operating parameters—temperature and activating gas flow rate—on product yield, mass loss, and the porous properties of the final product. In addition, comparative Aspen Plus simulation studies were conducted to support the experimental results and to thermodynamically compare the performance of each pure activating agent with that of their mixture.

Despite extensive research on physical activation using individual agents such as CO_2_, steam, or air, studies investigating the synergistic coupling of CO_2_ and air in a controlled fluidized-bed reactor remain limited. Most previous works focus either on single-agent activation, on combinations of more common activating agents such as H_2_O and CO_2_ applied simultaneously or successively, or on adsorption performance evaluation. However, the fundamental interaction between endothermic CO_2_ gasification and exothermic partial oxidation in terms of energy balance, activation severity, and pore evolution has not been comprehensively analyzed under the specific conditions used in this work. In particular, the thermodynamic implications of combining these activation agents and their influence on process energy efficiency have received little attention. Therefore, this study aims to bridge this gap by experimentally and thermodynamically investigating CO_2_–air co-activation of industrial gasification-derived biochar in a laboratory-scale fluidized-bed reactor.

## 2. Results and Discussion

### 2.1. Effect of Activation Conditions on Yield and Carbon Conversion

**Figure 1:**
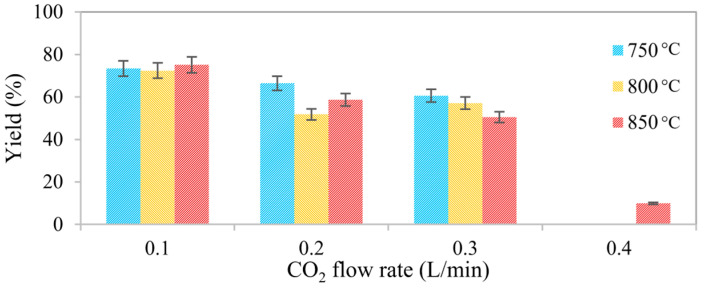
Effect of activation temperature and CO_2_ flow rate on yield. The air flow rate was adjusted proportionally to maintain a constant volumetric CO_2_/O_2_ ratio of 50:50 in all experiments. Activation time was fixed at 20 min for all conditions. Data at 0.4 L/min are available only at 850 °C; experiments at 750 and 800 °C were not included in the experimental design.

Figure 1 shows the effect of activation temperature and CO_2_ flow rate on the yield of activated biochar while the CO_2_–O_2_ ratio and activation time were kept constant. Increasing either the activation temperature or the CO_2_ flow rate led to a systematic decrease in yield, indicating enhanced carbon conversion and mass loss. The highest yields were obtained at 750 °C, whereas samples activated at 800 and 850 °C exhibited significantly lower yields, particularly at high CO_2_ flow rates. At the most severe conditions investigated (850 °C and 0.4 L/min CO_2_), the yield decreased to approximately 10%, indicating near-complete burnout of the carbonaceous structure. In contrast, the mildest conditions (750 °C and 0.1 L/min CO_2_) produced limited activation, with minimal influence of gas flow rate on conversion. Because 0.4 L/min was included only at 850 °C as a high-severity boundary case, Figure 1 does not contain corresponding points at 750 and 800 °C.

A comparison of the data obtained at 800 and 850 °C under intermediate CO_2_ flow rates (0.2 and 0.3 L/min) shows relatively comparable yield values. This suggests that, once a sufficient temperature and gas flow rate are reached to supply the required activation energy, further increases in temperature have a limited additional effect on carbon conversion. Under these conditions, enhanced gas availability promotes gas–solid interactions and accelerates carbon gasification. Minor deviations from the expected trends observed at 800 and 850 °C and CO_2_ flow rates of 0.2–0.3 L/min may be attributed to normal experimental uncertainties, including sample heterogeneity and variations in gas–solid contact, which become more noticeable when differences in mass loss and yield are small. These effects highlight the inherent complexity of activation kinetics, which involve overlapping processes such as devolatilization, gasification, pore development, and localized thermal fluctuations.

In addition to yield and mass loss, the porous properties of the activated biochar were also characterized. These textural parameters provide further insight into the activation process and offer more comprehensive criteria for selecting optimal operating conditions for biochar gasification and activation.

### 2.2. Development of Porous Structure and Textural Properties

**Figure 2:**
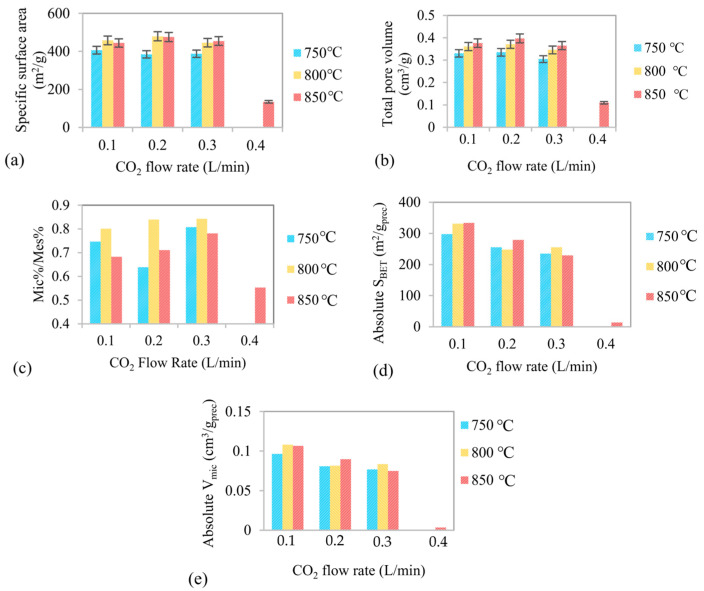
Effect of activation temperature and CO_2_ flow rate on the porous properties of activated biochar: (**a**) BET specific surface area, (**b**) total pore volume, (**c**) the ratio of microporosity percentage to the mesoporosity percentage, (**d**) Absolute BET surface area and (**e**) absolute micropore volume (bars, right axis) of activated biochar ($\mathrm{Absolute}\;S_{BET}(m^{2}/g_{prec})\;=\;S_{BET}\;\times \;(1\;-\;\mathrm{mass}\;\mathrm{loss})$, $\mathrm{Absolute}\;V_{mic}({cm}^{3}/g_{prec})=V_{mic}\;\times \;(1-\mathrm{mass}\;\mathrm{loss})$), The air flow rate was adjusted proportionally to maintain a constant volumetric CO_2_/O_2_ ratio of 50:50 in all experiments. The activation time was fixed at 20 min for all conditions.

Figure 2a–c illustrates the effects of CO_2_ flow rate and activation temperature on the textural properties of the activated biochar, including the BET specific surface area (S_BET_), total pore volume (V_tot_), and the ratio of microporosity percentage to mesoporosity percentage.

The evolution of S_BET_ with CO_2_ flow rate exhibits a similar trend at all temperatures, particularly at sufficiently high activation temperatures. In general, S_BET_ initially increases with increasing CO_2_ flow rate, reflecting enhanced pore development due to intensified gas–solid interactions. However, when the CO_2_ flow rate exceeds an optimal level, S_BET_ decreases because of excessive gasification. This deterioration of the porous structure is particularly evident at a CO_2_ flow rate of 0.4 L/min and an activation temperature of 850 °C, where significant destruction of the carbon framework is observed (Figure 2a,b). A similar increasing–decreasing trend is also observed for the total pore volume, indicating that pore generation and pore collapse occur sequentially as activation severity increases.

The highest S_BET_ and total pore volume were obtained for biochar activated at a CO_2_ flow rate of 0.2 L/min at 800 and 850 °C, respectively. At 800 °C, S_BET_ and V_tot_ reached 479.2 m^2^/g and 0.371 cm^3^/g, respectively, while at 850 °C comparable S_BET_ values (475.3 m^2^/g) were achieved with a higher total pore volume of 0.397 cm^3^/g. These results indicate that both temperatures can effectively promote pore development under moderate CO_2_ flow conditions.

The highest S_BET_ was obtained at 800 °C and a flow rate of 0.2 L/min. Increasing the temperature to 850 °C caused a decrease in S_BET_ alongside an increase in total pore volume, indicating that this temperature is too aggressive and leads to micropore collapse. This is confirmed by the Mic/Mes ratio in Figure 2c, which increases from 750 to 800 °C but decreases consistently from 800 to 850 °C. The same trend is observed at all flow rates. Thus, the decrease in S_BET_ and simultaneous increase in total pore volume between 800 and 850 °C can be attributed to the collapse and merging of micropores into larger meso- or macropores, which contribute less to the specific surface area.

Higher temperatures and CO_2_ flow rates intensify oxidation, increasing oxygen availability, accelerating carbon consumption, and promoting excessive pore widening and wall thinning. This can lead to structural instability and reduced surface area. These results highlight the importance of controlled partial oxidation in promoting micropore formation while maintaining structural integrity, consistent with recent reports on mild oxidative environments regulating carbon structure evolution. While mild oxidation promotes micropore development and increases surface area, excessive oxidation can destabilize the carbon framework, leading to pore widening, collapse, and degradation phenomena like those reported for oxidized carbon supports under electrochemical conditions [25].

The precursor biochar exhibited a BET specific surface area of approximately 313 m^2^/g and a total pore volume of 0.278 cm^3^/g. Comparison with the activated samples demonstrates that CO_2_/air gasification leads to a substantial enhancement of textural properties. Under optimized activation conditions, an average increase of approximately 50% in BET specific surface area and 30–35% in total pore volume was achieved relative to the precursor.

In addition to overall pore development, the relative contributions of microporosity and mesoporosity were evaluated. The ratio of microporosity percentage to mesoporosity percentage (Mic/Mes) under different activation conditions is presented in Figure 2c. This ratio provides direct insight into the balance between micropore formation and mesopore development as a function of activation severity.

In all cases, the Mic/Mes ratio remains below unity, indicating that mesopores constitute the dominant pore type in the activated biochar. However, at moderate CO_2_ flow rates of 0.2–0.3 L/min, particularly at 800 °C, the Mic/Mes ratio reaches its highest values (approximately 0.8–0.85). This behavior reflects favorable activation conditions that promote efficient micropore generation while limiting excessive mesopore widening. In contrast, at 850 °C under aggressive CO_2_ activation conditions, the porous structure can shift from predominantly microporous to larger pore domains due to over-activation. This process leads to micropore destruction, uncontrolled pore widening, and pore coalescence, thereby reducing the micropore/mesopore ratio. A similar phenomenon was reported by Phothong et al. [26] during the CO_2_ activation of bamboo biomass, where excessive activation severity (temperatures above ~950 °C) resulted in a decrease in micropore volume and overall porous properties due to the merging of neighboring pores and the loss of microporosity, with a corresponding increase in mesoporosity [26].

Under the optimal activation conditions (0.2–0.3 L/min and 800 °C), the pore structure consists of approximately 42–43% micropores and 50–51% mesopores. This balanced micro–mesoporous structure corresponds to the highest surface area and favorable pore accessibility, confirming these conditions as optimal for producing high-quality activated biochar.

**Figure 3:**
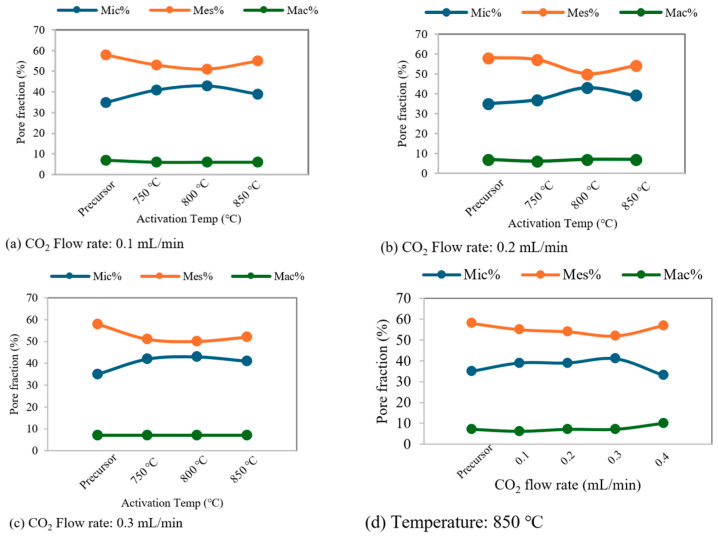
Pore fraction evolution of activated biochars as a function of activation temperature ((**a**–**c**), fixed CO_2_ flow rates) and CO_2_ flow rate ((**d**), fixed temperature at 850 °C).

The pore fraction evolution of activated biochar as a function of activation temperature (at fixed CO_2_ flow rates) and CO_2_ flow rate (at a fixed temperature of 850 °C) is also shown in Figure 3. The percentage of macropores remains relatively constant in most cases, except at the highest CO_2_ flow rate of 0.4 mL/min, where the activation conditions become particularly severe, especially at 850 °C. In addition, these plots clearly illustrate the evolution of micropore and mesopore contributions. The increase in micropore fraction as the temperature rises from 750 to 800 °C is accompanied by a decrease in mesopore contribution, indicating micropore development under mild oxidation conditions. At higher temperatures, however, the trend shifts toward increased mesopore contribution and partial destruction or widening of micropores, which supports the previously proposed mechanism.

In biochar gasification and activation studies, textural properties such as BET surface area and micropore volume are typically reported on a mass-normalized basis (per gram of product), which reflects only the intrinsic porosity of the remaining solid and does not account for material loss during activation. However, increasing activation severity usually causes significant yield reduction while improving porosity, making it difficult to evaluate the overall efficiency of the process based solely on mass-normalized values. Therefore, absolute BET surface area and absolute micropore volume are introduced as complementary indicators to assess the true mass efficiency of activation. These absolute textural parameters were calculated by correcting the measured BET surface area and micropore volume with the remaining mass fraction after activation.

Figure 2d,e show the variation in absolute BET surface area and absolute micropore volume with CO_2_ flow rate and activation temperature. These parameters represent the net surface area and microporosity retained relative to the initial biochar mass and thus reflect the balance between pore development and carbon loss. The highest absolute values are observed under mild activation conditions (800 °C and 0.1 L/min), primarily due to moderate burn-off and pore generation. As activation severity increases, intrinsic porosity (S_BET_ and V_Mic_) initially improves; however, increasing carbon loss progressively offsets this enhancement, leading to a decline in the absolute values. Under severe conditions (850 °C and 0.4 L/min), both absolute parameters decrease sharply, indicating that excessive gasification causes structural collapse and loss of net porosity.

In the present study, severe activation conditions reduce both yield and intrinsic porosity, as excessive gasification leads to pore destruction rather than productive pore formation. Consequently, absolute textural parameters are not used as primary criteria for determining optimal activation conditions. Instead, optimization is based on intrinsic textural properties and pore structure balance, while absolute parameters serve to confirm the inefficiency of overly severe activation.

### 2.3. Evolution of Proximate Composition and Carbon Framework Stability

**Figure 4:**
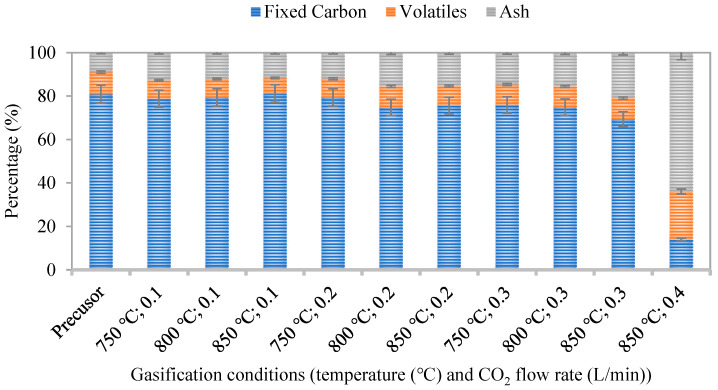
Proximate composition (fixed carbon, volatile matter, and ash) of the precursor and activated biochar produced under different activation temperatures and CO_2_ flow rates. The air flow rate was adjusted proportionally to maintain a constant volumetric CO_2_/O_2_ ratio of 50:50 in all experiments. The activation time was fixed at 20 min for all conditions.

In addition to yield, mass loss, and textural properties, the proximate composition of the activated biochar was analyzed to determine the distribution of fixed carbon (FC), volatile matter, and ash. Figure 4 presents the proximate composition of biochar produced under different activation temperatures and CO_2_ flow rates. The precursor biochar contained approximately 80 wt.% fixed carbon, 12 wt.% volatile matter, and 8 wt.% ash.

The results show a systematic decrease in fixed carbon content with increasing activation severity, accompanied by a pronounced increase in ash fraction, particularly at high temperatures and CO_2_ flow rates. While volatile matter decreases mainly during the initial stages of activation and remains relatively stable thereafter, fixed carbon is progressively consumed through gasification, leading to carbon depletion and mineral enrichment. Under extreme conditions (850 °C and 0.4 L/min), near-complete carbon burnout occurs, producing ash-dominated residues and explaining the severe deterioration of textural properties observed under these conditions.

In contrast, mild activation conditions (750 °C and low CO_2_ flow rates) preserve a high fraction of fixed carbon with only minor changes in ash content. Under the optimized activation conditions (800 °C and 0.2–0.3 L/min CO_2_), the fixed carbon content decreases moderately from about 80.9 wt.% in the precursor to 74.8–75.0 wt.% in the activated biochar, corresponding to an FC reduction of approximately 7%. This limited carbon consumption indicates controlled gasification that is sufficient for pore development while maintaining the carbon framework. Overall, these results highlight the importance of carefully controlling activation severity, as excessive conditions lead to structural degradation and ash accumulation, whereas moderate conditions favor the production of high-quality activated biochar.

Although adsorption testing was not part of the original scope of this work, the porous characteristics obtained in this study (BET surface area of 479 m^2^/g with approximately 42% microporosity) provide a useful basis for comparison with previously reported adsorbents. The achieved surface area is higher than that of many physically activated biochars reported in the literature, which can be attributed both to the relatively high initial surface area of the precursor biochar [27] and to the effectiveness of the CO_2_–air activation process in developing pore structure.

A review of the literature shows that CO_2_-activated wheat bran biochar, with a surface area of approximately 340–347 m^2^/g and a predominantly microporous structure, demonstrated methylene blue adsorption capacities in the range of 150–241.95 mg/g at 303 K [28]. In addition, Fang et al. investigated CO_2_-activated hickory hydrochars with BET surface areas ranging from approximately 400 to 670 m^2^/g and reported enhanced adsorption performance toward methylene blue and heavy metal ions (Pb^2+^, Cu^2+^, Cd^2+^), showing that adsorption capacity increased with activation severity and pore development [29].

This comparison suggests that the adsorbent produced in this study is expected to exhibit acceptable sorption capacity toward organic molecules and metal cations from wastewater, owing to its meso-microporous structure and the oxygen functionalities likely introduced during CO_2_ and air activation.

### 2.4. Thermodynamic Interpretation of Activation Atmospheres

To provide deeper insight into the behavior of different activating agents in the absence of direct experimental gas-composition data, equilibrium simulations were performed using Aspen Plus software V14.0 with the RGIBBS reactor model. Equilibrium simulations using Gibbs minimization are commonly applied to predict syngas composition and performance in biomass and carbonaceous feedstock gasification systems [30,31]. Biochar was approximated as graphite (pure carbon) to focus on the primary gasification reactions, while neglecting secondary effects of ash and surface functionalities. The simulations were conducted within the experimental temperature window (750–850 °C) and total molar gas flow rates scaled to laboratory levels (equivalent to approximately 0.1–0.4 L/min in the experiments). Three activation atmospheres were compared under identical total flow rates: CO_2_ only, air only, and a CO_2_–air mixture (volumetric O_2_/CO_2_ ratio of 1:1).

The simulations consistently predict CO as the dominant gaseous product across all atmospheres, in agreement with thermodynamic expectations at elevated temperatures. Both the endothermic Boudouard reaction (C + CO_2_ ⇌ 2CO, ΔH ≈ +172 kJ/mol) and the exothermic partial oxidation (C + ½O_2_ ⇌ CO, ΔH ≈ -111 kJ/mol) favor CO formation above 700 °C, while full combustion to CO_2_ remains limited under equilibrium conditions [6,32].

The results clearly illustrate the synergistic advantages of the CO_2_–air mixture. CO_2_-only activation remains consistently endothermic, with heat duties ranging from approximately +0.067 to +0.013 kW as total flow increases, reflecting the intrinsically energy-intensive nature of CO_2_ gasification. Air-only activation exhibits higher initial yields but transitions to strongly exothermic behavior at elevated flows and temperatures (–0.12 to –0.13 kW), highlighting the aggressive reactivity of O_2_ and the risk of uncontrolled burnout.

The CO_2_–air mixture exhibits an intermediate and highly advantageous thermodynamic behavior. At low flow rates the system remains mildly endothermic (+0.13 kW), while at moderate and high flows it becomes exothermic (−0.02 to −0.10 kW), indicating that the exothermic heat released by partial oxidation can partially or fully compensate the endothermic demand of CO_2_ gasification. Compared with CO_2_-only activation, the mixed atmosphere substantially reduces the net external energy requirement while preserving intermediate carbon yields (approximately 16–58%), thereby providing an intrinsic mechanism for thermal self-sustaining of the activation process. This thermodynamic tuning supports the experimental findings of optimal pore structure and surface area at moderate activation severity.

A particularly important outcome of the simulations is that total oxidant supply (i.e., gas flow rate) exerts a significantly stronger influence on carbon conversion than temperature within the investigated range. Increasing total flow drives carbon yield from 58 to 66% to near complete conversion, whereas increasing temperature from 750 to 850 °C at fixed flow produces only minor yield variations (generally <5%). Temperature mainly enhances CO selectivity and slightly improves thermal efficiency in endothermic-dominant regimes but does not govern conversion to the same extent as oxidant availability. Like the present findings, Reyes et al. [33] demonstrated that increasing CO_2_ partial pressure and CO_2_/C ratio significantly accelerates biochar conversion and reduces the time required for complete gasification, highlighting the dominant influence of oxidant supply on carbon consumption [33].

Although real activation processes do not reach thermodynamic equilibrium due to kinetic limitations, finite residence time, and mass-transfer constraints, these non-ideal effects lead to experimental carbon yields (10–75%) that differ from those predicted by equilibrium calculations (16–58%). This discrepancy between equilibrium-predicted yields and experimentally measured values appears to be systematic: under more severe operating conditions, the experimental yields are lower than the equilibrium-estimated values. The lower experimental yields relative to simulations may be attributed to local overheating, pore collapse leading to a sharp reduction in specific surface area, or excessive burn-off caused by non-ideal flow patterns. In contrast, under mild operating conditions, the higher experimental yields compared to simulations may result from kinetic limitations, finite residence time, or mass-transfer resistance preventing the system from reaching complete gasification equilibrium.

Nevertheless, the simulations still provide robust thermodynamic benchmarks. They confirm that combining CO_2_ and air enables direct coupling of exothermic and endothermic reactions, creating a thermodynamically favorable activation environment that explains the experimentally observed balance between high surface area development and controlled burn-off.

Similar equilibrium-based Aspen Plus analyses of biomass gasification report dominant CO formation, strong dependence on oxidant supply, and the usefulness of thermodynamic models for interpreting experimental trends, despite deviations caused by kinetic and transport limitations [30].

While Aspen-based thermodynamic modeling provides valuable insight into relative heat demands and feasible heat-integration strategies, a comprehensive techno-economic analysis (TEA) and life-cycle assessment (LCA) are recommended to fully evaluate process sustainability, including utility consumption, gas handling, yield losses, and emissions [34,35,36].

## 3. Materials and Methods

### 3.1. Raw Materials and Pretreatment

The powdered biochar used in this study as the raw material originates from a floating fixed bed gasification reactor developed by SynCraft^®^ GmbH (Schwaz, Austria) and operated by Innsbrucker Kommunalbetriebe AG (IKB, Innsbruck, Austria). The feedstock for this reactor consisted primarily of forest wood chips, which were first dried, then autothermally pyrolyzed, and subsequently gasified. Details of this process can be found elsewhere [37].

Prior to activation, the raw biochar was characterized by proximate analysis, and it contains approximately 80 wt.% fixed carbon, 12 wt.% volatile matter, and 8 wt.% ash. BET analysis indicated a specific surface area of 313 m^2^/g and a total pore volume of 0.278 cm^3^/g, with a pore distribution of 34% micropores, 59% mesopores, and 7% macropores.

**Figure 5:**
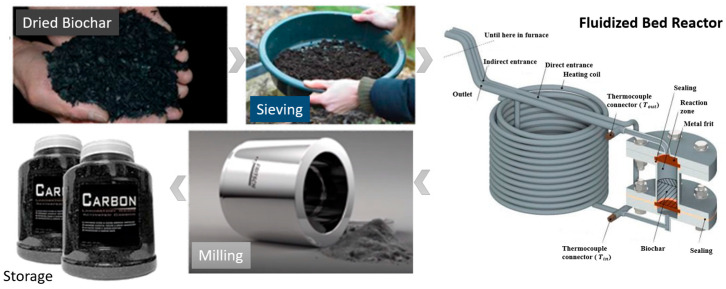
Steps of the biochar preparation process and the laboratory-scale fluidized bed reactor used for activation [18].

The gasified biochar undergoes several preparation steps before activation (Figure 5), including drying at 105 °C for 24 h, cooling in a desiccator, and screening through a 2 mm sieve to separate coarse particles (∼50%). To ensure a uniform particle size distribution and maximize the usable fraction of biochar, the coarse fraction is milled for 10 min at 400 rpm using a planetary ball mill. The fine fraction and the milled coarse fraction are then blended and stored in a hermetically sealed Schott Duran bottle to ensure consistency for subsequent activation experiments.

### 3.2. Activation Reactor and Essential Preprocessing Steps

**Figure 6:**
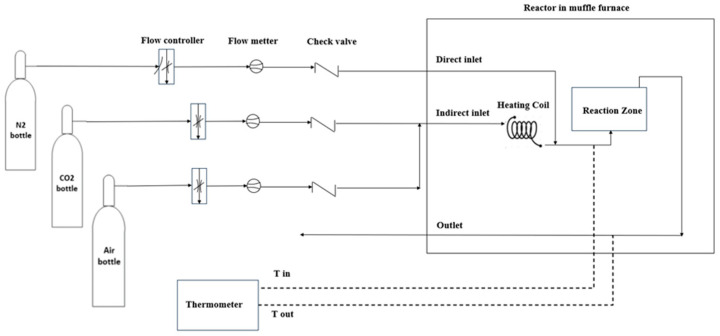
Flowchart illustrating the gas delivery system and reactor setup, including direct/indirect inlets, heating coil, reaction zone, and temperature sensors.

The activation experiments were carried out in a laboratory-scale vertical tubular reactor system described in detail elsewhere [18]. The reactor had an internal diameter of 34 mm and a height of 50 mm, corresponding to a total volume of approximately 45 mL, with the reaction zone occupying roughly one-third of the reactor length and containing 7 g of biochar. Two gas inlets were used: a direct inlet for heating and cooling and an indirect inlet that routed the activating gases through a preheated coil during activation. A metal frit at the reactor base ensured uniform gas distribution, while a second frit at the outlet prevented particle entrainment. A schematic of the setup is shown in Figure 6.

Before each experiment, the reactor was assembled with intact seals and checked for leak-tightness using nitrogen. The reactor was then placed in a preheated muffle furnace (Nabertherm B150, New Castle, DE, USA), heated to the target activation temperature, and operated under the activation conditions described in Section 3.3. Once the target temperature was reached, the gas supply was switched from nitrogen to the CO_2_–air mixture through the indirect inlet to initiate activation. After the designated activation period, the furnace was shut down, and nitrogen was reintroduced via the direct inlet (2 mL/min) during cooling to prevent oxidation. Following cooling to room temperature, the activated biochar was recovered, weighed, and stored for further analysis.

### 3.3. Activation Experiments

A mixture of CO_2_–air was used as the activating agent. The initial CO_2_/O_2_ ratio of 50/50 (*v*/*v*) was selected based on the findings of Hanaoka et al. [19], who demonstrated that this composition enhances char reactivity under N_2_/CO_2_/O_2_ atmospheres during char production and its CO_2_ gasification behavior [19].

The CO_2_ and air flow rates were adjusted to achieve equivalent molar proportions of CO_2_ and O_2_ in the activation stream. In their study, a gas composition of approximately 18/41/41 vol.% (N_2_/CO_2_/O_2_), representing a near 1:1 CO_2_/O_2_ ratio within the oxidizing fraction, resulted in the highest BET specific surface area (417 m^2^/g) and the largest pore volume (0.24 cm^3^/g). Moreover, the gasification rate constant (K_p_) at 900 °C under this composition was 1.7–2.5 times higher than under other N_2_/CO_2_/O_2_ ratios, indicating a synergistic balance between partial oxidation and CO_2_ gasification. Based on these findings, the present work adopted a fixed 50:50 CO_2_/O_2_ ratio to ensure operation within a literature-supported optimal regime [19].

The activation experiments were conducted by varying two main operating factors: temperature and gas flow rate. Three temperatures (750, 800, and 850 °C) were tested, while the activation time was kept constant at 20 min to enable systematic comparison of temperature and gas flow rate effects while maintaining a consistent reaction time across all experiments. This duration aligns with previous optimization studies on the physical activation of gasification-derived char, which report representative and near-optimal residence times in the range of approximately 15–24 min. Furthermore, these studies indicate that prolonged activation under high-severity conditions may lead to over-activation and structural degradation, supporting the selection of 20 min as a controlled and technically relevant duration.

The full factorial design included CO_2_ flow rates of 0.1–0.3 L/min at all temperatures (750–850 °C). In addition, a higher CO_2_ flow rate of 0.4 L/min was evaluated exclusively at 850 °C as a high-severity boundary condition to examine the onset of over-activation and potential framework degradation. For CO_2_ flow rates of 0.1, 0.2, and 0.3 L/min, the corresponding air flow rates (0.47, 0.95, and 1.42 L/min, respectively) were adjusted to maintain a constant volumetric CO_2_/O_2_ ratio of 50:50.

Each parameter set was tested in duplicate, and when the standard deviation exceeded the acceptable threshold (5%), additional runs were performed to ensure data reliability and reproducibility.

### 3.4. Characterization

The gasified activated biochar produced in this study was characterized using thermogravimetric analysis (TGA) and N_2_ adsorption measurements. TGA was used to determine the proximate composition (volatile matter, fixed carbon, and ash) of both the precursor and activated biochar in accordance with DIN 51720:2001-03 [38]. Measurements were performed using a Mettler Toledo TGA/DSC 1 STARe system with a constant gas flow rate of 100 mL/min. The samples were heated from 30 to 900 °C under a nitrogen atmosphere to quantify the volatile matter. Afterward, the atmosphere was switched to synthetic air at 900 °C to combust the remaining carbonaceous material, allowing determination of the fixed carbon content, while the residual mass was taken as the ash content. This stepwise heating procedure enabled reliable determination of the proximate composition of the biochar.

Gas adsorption measurements were carried out to characterize the textural properties of the activated carbons, including the specific surface area, total pore volume, and pore size distribution. Nitrogen adsorption–desorption experiments were performed using a Micromeritics 3Flex surface characterization analyzer. Prior to analysis, the samples were degassed ex situ using a VacPrep 061 unit to remove residual moisture and adsorbed gases. Degassing was conducted under vacuum at approximately 0.04 mbar and 200 °C for 24 h, which typically resulted in a mass loss of about 4%. The pore size distribution was calculated using the non-local density functional theory (NLDFT) method, applying the “Het N_2_ Carbon Slit” model to account for slit-shaped pores commonly found in carbon materials.

## 4. Conclusions

CO_2_–air co-activation in a fluidized-bed reactor enables the energy-efficient production of high-quality biochar with controlled porosity. Based on the experimental results, activation severity—reflected by solid yield and carbon conversion—is governed primarily by gas flow rate, while temperature plays a secondary role within the investigated range of 750–850 °C. In contrast, the development of textural properties such as BET surface area (S_BET_) and total pore volume (V_tot_) is controlled by the combined influence of flow rate and temperature, exhibiting distinct optima rather than monotonic trends. The optimal operating window for achieving the highest porosity was identified at 800 °C and a CO_2_ flow rate of 0.2–0.3 L/min. The maximum BET surface area (479 m^2^/g) was obtained at a CO_2_ flow rate of 0.2 L/min, accompanied by a reasonable total pore volume of 0.37 cm^3^/g. Thermodynamic equilibrium simulations in Aspen Plus corroborate the experimental trends and demonstrate that CO_2_–air co-activation establishes a near-thermoneutral to mildly exothermic activation environment (≈+0.13 to −0.10 kW), in contrast to strongly endothermic CO_2_-only activation (up to +0.07 kW) and strongly exothermic air activation (down to −0.13 kW). This intrinsic coupling of endothermic CO_2_ gasification with exothermic partial oxidation reduces external heat demand by approximately 60–70% and improves process stability.

## Data Availability

The data presented in this study are available within the article. Additional data supporting the findings of this study are available from the corresponding authors upon reasonable request.

## Abbreviations

The following abbreviations are used in this manuscript:

TGA	Thermogravimetric analysis
TGFBR	Thermogravimetric fluidized bed reactor
MCI	Management Center Innsbruck
S_BET_	BET specific surface area
V_tot_	Total pore volume
Mic	Microporosity
Mes	Mesoporosity
FC	Fixed carbon
TEA	Techno-economic analysis
LCA	Life-cycle assessment
IKB	Innsbrucker Kommunalbetriebe AG
NLDFT	Non-local density functional theory
prec	precursor
